# Telehealth for Care of People in the Brazilian Primary Healthcare: An Integrative Review

**DOI:** 10.1002/puh2.70438

**Published:** 2026-11-13

**Authors:** Márcio Cristiano de Melo, Ivana Daniela César, Elizabeth Regina de Melo Cabral, Brenda Leandro dos Santos, Lívia Maria Ferrante Vizzotto Consoli, Rinaldo Eduardo Machado de Oliveira

**Affiliations:** ^1^ São Leopoldo Mandic School of Medicine Araras São Paulo Brazil; ^2^ University of Brasília Brasília Federal District Brazil; ^3^ Ribeirão Preto Medical School University of São Paulo Ribeirão Preto São Paulo Brazil

**Keywords:** access to primary care, comprehensive healthcare, digital public health, eHealth policies, telemedicine

## Abstract

**Background and Aim:**

Telehealth is a care model in primary health care (PHC), especially in hard‐to‐reach areas where the shortage of health professionals is still a problem. The aim is to know and synthesize the articles on experiences with telehealth in the Brazilian PHC.

**Methods:**

This is an integrative review in which bibliographic databases were searched for articles published in Portuguese, English or Spanish between 2014 and 2024.

**Results:**

Twenty articles were selected, all being observational. It was found that six studies (30%) were conducted in the PHC of the state of Minas Gerais, five (25%) in the state of Rio Grande do Sul, and four (20%) reported the domestic scenario. Teleconsulting was the most frequently reported telehealth modality across the included studies (60%), predominantly involving provider‐to‐provider interactions aimed at clinical support and decision‐making. Direct provider‐to‐patient remote consultation was rarely reported in the included studies. No studies addressing experiences with remote consultation for follow‐up and education of patients with non‐communicable chronic conditions were found, nor teleservices by multi‐professional teams. Studies on teleconsultation for mental healthcare, a frequent demand in PHC, were not observed either.

**Conclusion:**

Although telehealth initiatives have been reported in different regions of the country, the available evidence is concentrated in specific states, particularly in the Southeast and South regions; there are distinctions, especially those related to infrastructure. In addition, no studies addressing telehealth interface with attributes derived from PHC were found, such as cultural competence as well as family and community orientation.

## Graphical abstract

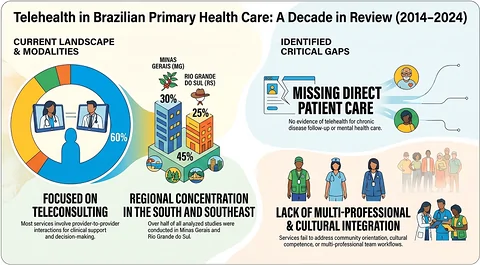

## 1 Introduction

Telehealth for the care of people attending the primary healthcare (PHC) is notorious in the Unified Health Systems (SUS) [1]. Although countries, like the United States and Canada, adopt telehealth to overcome geographical barriers, in Brazil, it has been seen as a fundamental model for PHC, especially in difficult‐to‐reach areas where the shortage of health professionals is still a critical problem [1, 2]. The SUS, whose PHC serves as the preferential gateway, has been facing persistent challenges related to the inequality of access to healthcare services, especially in remote and socially vulnerable regions. In this context, digital transformation in the SUS by means of telehealth has been shown to be a viable strategy to mitigate such disparities [2, 3].

The COVID‐19 pandemic highlighted the urgency of telehealth in Brazil, leading to the enactment of law 13989/2020, which authorized in emergency situations [4]. During the health crisis, telehealth was shown to be an efficient model to provide health services remotely, thus reducing lines, optimizing resources and increasing access to care. Such an accelerated expansion, boosted by the necessity of social distancing, evidenced the potential of telehealth to transform the PHC model [5].

Studies have pointed out that the Brazilian Telehealth Network Program, which had already been under development, was strengthened with the integration of external digital technologies for health promotion [6]. Recent evidence suggests that the continuity of telehealth services during the post‐pandemic period is viable and desired if there is adequate regulation and sufficient technological infrastructure for professionals and patients [7].

Information and communication technologies (ICTs) play a crucial role in the health digital transformation by offering new possibilities of improving management, access to and quality of health services [8]. In the context of PHC, ICTs allow the use of tools such as electronic medical records, on‐line scheduling systems and teleconsultation platforms, thus promoting integration and collaboration among different health services. These technologies enable clinical data to be stored and shared efficiently, which improves the monitoring of the patients’ health and facilitates the continuity of care. In addition, the use of ICTs seeks to streamline the communication between healthcare teams, patients and managers [9].

In the telehealth context, ICTs are the basis for the functioning of platforms aimed at increasing the access to specialized professionals and reducing geographical barriers. These technologies facilitate real‐time information exchange by providing immediate clinical support and enabling permanent health education. The efficient use of ICTs in telehealth also allows for a more effective management of the SUS resources by optimizing the distribution of services, reducing costs and promoting more equality in the access to healthcare [7, 9].

The wide implementation of telehealth in Brazil has been facing challenges, such as unequal access to quality internet and lack of qualification of health professionals for use of digital platforms. Overcoming these obstacles depends on public policies aimed at ensuring digital inclusion and expanding telehealth beyond the major urban centres [5, 6, 9].

Long‐term sustainability of telehealth in Brazil needs integration between different levels of healthcare and available information systems. The creation of a telehealth network to allow a direct dialogue with programmes, such as the Family Health Strategy (FHS), is crucial to ensure that the generated information can be used on a coordinated basis. This will allow a continuous flow of clinical and operational data, thus increasing the monitoring and assessment of results, in addition to promoting the inclusion of remote areas in the national digital health network [10].

In view of the expansion of telehealth in Brazil and of its potential of digital transformation in the SUS, especially in the context of PHC, it is crucial to understand experiences and impacts [10, 11]. The analysis of the existing evidence can help in the formulation of public policies aimed at ensuring sustainability and expansion of telehealth, thus promoting digital inclusion and improving access to healthcare. In this context, the aim is to know and synthesize the articles on experiences with telehealth in the Brazilian PHC.

## 2 Methods

This integrative review was conducted following the methodological framework which comprises five sequential and interrelated stages: problem identification, literature search, data evaluation, data analysis and presentation of results [12]. Initially, the research problem and guiding question were clearly defined to delimit the scope of the review, followed by a comprehensive and systematic literature search across multiple databases using controlled descriptors and Boolean operators to ensure sensitivity and specificity. In the data evaluation stage, studies were selected according to predefined inclusion and exclusion criteria, and their methodological quality was critically appraised to support the interpretation of findings.

Data analyses were performed through a structured and iterative process involving data reduction, comparison and grouping of studies into thematic categories on the basis of telehealth modalities, target populations and reported outcomes, allowing the identification of patterns, consistencies and gaps in the literature. Finally, results were synthesized and presented in a narrative and tabular format to enhance clarity and transparency. In addition, the reporting of this review was guided by the Preferred Reporting Items for Systematic Reviews and Meta‐Analyses (PRISMA 2020) [13] statement, with adaptations to accommodate the integrative review design, ensuring transparency in the selection process, reproducibility of the search strategy and completeness in reporting the identification, screening, eligibility and inclusion of studies.

The present review was performed in October 2024 by searching the following bibliographic databases: *Biblioteca Virtual em Saúde (BVS*) (https://bvsalud.org/), *Literatura Latino‐Americana e do Caribe em Ciências da Saúde* (LILACS) (http://lilacs.bvsalud.org), MEDLINE via PubMed (https://www.ncbi.nlm.nih.gov/pmc/) and EBSCO (https://www.ebsco.com). The final search was conducted on 15 October 2024. Searches were performed in title, abstract and keywords fields when applicable. The descriptors of *Ciências da Saúde* (*DeCS*) or Medical Subject Headings (MeSH) were used. In English, they were ʻtelemedicineʼ (*DeCs* and MeSH), eHealth strategiesʼ (*DeCS*), ʻdigital healthʼ (*DeCS*), ʻvirtual medicineʼ (MeSH), ʻmobile healthʼ (MeSH), ʻmHealthʼ (MeSH), ʻtelehealthʼ (MeSH), ʻeHealth telecareʼ (MeSH), ʻtele careʼ (MeSH), ʻ“primary health care” (*DeCS* and MeSH), ʻcare, primary healthʼ (MeSH), ʻhealth care, primaryʼ (MeSH), ʻprimary careʼ (MeSH), ʻcare, primaryʼ (MeSH), ʻprimary healthcareʼ (MeSH), ʻhealthcare, primaryʼ (MeSH) and ʻBrazilʼ (*DeCS* and MeSH). In Spanish, they were ʻtelemedicinaʼ (*DeCS*), ʻsalud digitalʼ (*DeCS*), ʻatención primaria de saludʼ (*DeCS*) and ʻBrasilʼ (*DeCS*). In Portuguese, they were ʻtelessaúdeʼ, ʻtelemedicinaʼ, ʻserviço de telemedicinaʼ, ʻserviço de telessaúdeʼ, ʻtelecuidadoʼ, ʻteleassistênciaʼ, ʻestratégias de eSaúdeʼ, ʻsaúde digitalʼ, ʻatenção primária à saúdeʼ, ʻatenção básica à saúdeʼ, ʻcuidados primários à saúdeʼ and ʻBrasilʼ, all descriptors of *DeCS*.

Boolean operators AND and OR were used by always crossing one of the first three descriptors with one of the others. The search in English was performed in all databases, although LILACS and MEDLINE were also searched in Portuguese and Spanish, as these databases contain articles published in these languages. Therefore, the following search commands were defined for selection of the articles: Descriptors (*DeCS*) in Portuguese were ʻtelessaúdeʼ OR ʻtelemedicinaʼ OR ʻserviço de telemedicinaʼ OR ʻserviço de telessaúdeʼ OR ʻtelecuidadoʼ OR ʻteleassistênciaʼ OR ʻestratégias de eSaúdeʼ OR ʻsaúde digitalʼ AND ʻatenção primária à saúdeʼ OR ʻatenção básica à saúdeʼ OR ʻcuidados primários à saúdeʼ AND ʻBrasilʼ; descriptors (DeCS) in English were (ʻtelemedicineʼ OR ʻeHealth strategiesʼ OR ʻdigital healthʼ) AND (ʻprimary health careʼ) AND (ʻBrazilʼ); and descriptors (*DeCS*) in Spanish were ʻtelemedicinaʼ OR ʻsalud digitalʼ AND ʻatención primaria de saludʼ AND ʻBrasilʼ. Descriptors (MeSH) in English were ʻtelemedicineʼ OR ʻvirtual medicineʼ OR ʻmobile healthʼ OR ʻmHealthʼ OR ʻtelehealthʼ OR ʻeHealthʼ OR ʻtelecareʼ OR ʻtele careʼ AND ʻprimary health careʼ OR ʻcare, primary healthʼ OR ʻhealth care, primaryʼ OR ʻprimary careʼ OR ʻcare, primaryʼ OR ʻprimary healthcareʼ OR ʻhealthcare, primaryʼ AND ʻBrazilʼ.

The search strategy applied in MEDLINE via PubMed was structured using MeSH terms and free‐text terms combined with Boolean operators. The following search string was used: (ʻtelemedicineʼ[MeSH Terms] OR ʻtelemedicineʼ[Title/Abstract] OR ʻtelehealthʼ[Title/Abstract] OR ʻeHealthʼ[Title/Abstract] OR ʻdigital healthʼ[Title/Abstract] OR ʻmHealthʼ[Title/Abstract] OR ʻmobile healthʼ[MeSH Terms]) AND (ʻprimary health careʼ[MeSH Terms] OR ʻprimary careʼ[Title/Abstract] OR ʻprimary healthcareʼ[Title/Abstract] OR ʻhealth care, primaryʼ[MeSH Terms]) AND (ʻBrazilʼ[MeSH Terms] OR ʻBrazilʼ[Title/Abstract]). Searches were conducted in title, abstract and MeSH fields. Filters were applied to include studies published between 2014 and 2024, in English, Portuguese or Spanish.

The search strategy in LILACS, accessed through the Biblioteca Virtual em Saúde (BVS), was performed using DeCS descriptors and free‐text terms in Portuguese, Spanish and English. The following search string was applied: (tw:(telessaúde OR telemedicina OR telecuidado OR teleassistência OR ʻsaúde digitalʼ OR ʻestratégias de eSaúdeʼ)) AND (tw:(ʻatenção primária à saúdeʼ OR ʻatenção básica à saúdeʼ OR ʻcuidados primários à saúdeʼ)) AND (tw:(Brasil)). The search was conducted in title, abstract and subject fields, with filters for publication period (2014–2024) and languages (Portuguese, English and Spanish).

In the general BVS database, the search strategy combined DeCS descriptors and free‐text terms across multiple languages. The following string was used: (tw:(telemedicina OR telessaúde OR ʻdigital healthʼ OR eHealth OR mHealth)) AND (tw:(ʻatenção primária à saúdeʼ OR ʻprimary health careʼ OR ʻatención primaria de saludʼ)) AND (tw:(Brasil OR Brazil)). Searches were performed in title, abstract and subject fields, considering studies published between 2014 and 2024, in Portuguese, English or Spanish.

The search conducted in the EBSCO database used free‐text terms combined with Boolean operators, focusing on title and abstract fields. The following search string was applied: (telemedicine OR telehealth OR eHealth OR ʻdigital healthʼ OR mHealth) AND (ʻprimary health careʼ OR ʻprimary careʼ OR ʻprimary healthcareʼ) AND (Brazil). Filters were applied to restrict results to studies published between 2014 and 2024, in English, Portuguese or Spanish.

The selection period was from 2014 to 2024. Only articles addressing the proposed theme according to the guiding question, published in Portuguese, Spanish and English and which could be fully read on‐line were included for review. Incomplete articles with only abstract available, articles on violence in other populations, theses and dissertations, letters to the editor, literature reviews and articles not answering the guiding question were excluded.

The methodological quality of included studies was assessed using the Mixed Methods Appraisal Tool (MMAT), version 2018 [14]. In this study, Section 4 of the instrument, designed for observational studies, was used. Initially, the two mandatory screening questions were addressed. Subsequently, the articles were evaluated on the basis of five criteria: clarity regarding participants, precision of measurements, completeness of outcome data, control of confounding variables and fidelity to the intended intervention or exposure. The evaluation was conducted independently by two reviewers. Disagreements were resolved through consensus or by a third reviewer. The instrument's authors advise against calculating a numerical score; therefore, articles meeting most of the criteria and qualitatively classified as being of moderate quality were included.

Data were analysed using thematic content analysis. Studies were grouped into predefined categories on the basis of telehealth modality (teleconsulting, teleeducation and telediagnosis), target population (professionals vs. patients) and type of outcome (access, quality, satisfaction, resolution). Categories were iteratively refined during analysis.

After excluding duplicates, titles and abstracts were read separately by two evaluators. If the article was considered eligible for inclusion, they were fully read. After the articles were read twice, any discordance for inclusion or exclusion was solved by a third evaluator. These data can be ordered through the corresponding author.

The screening process was conducted independently by two reviewers using a standardized form. Prior to screening, a calibration exercise was performed to ensure consistency. Disagreements were resolved through discussion and, when necessary, by a third reviewer. Although formal inter‐rater agreement statistics were not calculated, consensus procedures were systematically applied throughout the selection process. The present review study assessed articles available in free‐access databases with no limitation for privacy, security or access control. Therefore, no submission to the local research ethics committee was necessary [14].

## 3 Results

Twenty articles were included according to the eligibility criteria for the present review. Figure 1 shows a flowchart of the selection process. The methodological quality of the included studies, assessed using the Mixed Methods Appraisal Tool (MMAT), version 2018 [14], was predominantly moderate, reflecting the observational and descriptive nature of the evidence base. Most studies met key criteria related to clarity of objectives, adequacy of data collection methods and consistency of reported findings. However, important limitations were identified, particularly regarding sampling strategies, control of confounding variables and external validity, which should be considered when interpreting the results of this review.

**FIGURE 1:**
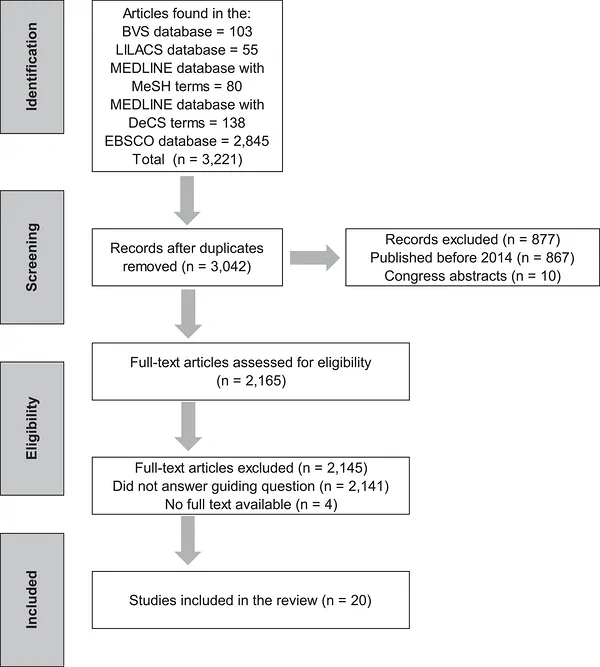
PRISMA 2020 adapted flow diagram illustrating the study selection process for the integrative review on telehealth in Brazilian primary healthcare (PHC), covering the period from 2014 to 2024.

Box 1 presents the included articles, with 12 (60%) being published between 2020 and 2024. All the articles (100%) were observational studies reporting aspects of telehealth in the PHC. It was found that six (30%) studies were conducted in the PHC of the state of Minas Gerais (MG), five (25%) in the state of Rio Grande do Sul (RS) and four (20%) focused on the Brazilian scenario. Teleconsulting was the most frequently reported telehealth modality across the included studies (60%), predominantly involving provider‐to‐provider interactions aimed at clinical support and decision‐making. Direct provider‐to‐patient remote consultation was rarely reported in the included studies, followed by teleeducation for PHC professionals (10%). Only one study (5%) showed the results of synchronous remote consultation between teleconsulting physician, patient and local physician in the PHC of a riverside district in Santarem, state of Pará.

**BOX 1:** Characterization, objectives/questions for investigation of studies, type and locality of the study, population/sample and main results of telehealth for care of people in the Brazilian primary healthcare (PHC) during the period from 2014 to 2024.

Identification	Title of the article and year of publication	Objective/Question for investigation	Type of study	Local of the study	Population/Sample	Main results
1	Doubts about the Diagnosis and Treatment of Syphilis in Pregnancy among Primary Care Professionals in a Telehealth Service [15] (2024)	To describe doubts about diagnosis and treatment of syphilis in pregnancy among primary care professionals in a telehealth service	Cross‐sectional	PHC in the state of RS	*n* = 356 teleconsultations provided by TelessaúdeRS‐UFRGS from 2018 to 2021 for PHC services involving syphilis in pregnancy	− The health metropolitan macro‐region accounted for 72% of the teleconsultations − The main doubts were related to the need for re‐treatment (35%), diagnostic definition (23%) and initial treatment (16%) − Maintenance of PHC follow‐up was suggested by 98.6% of the teleconsultations
2	Reasons for Provider‐to‐Provider Synchronous Teleconsultations Between Nurses in Primary Care: A Cross‐Sectional Study of TelessaúdeRS‐UFRGS Service [16] (2024)	To explore the results of a synchronous teleconsultation service for professionals and nurses in Brazil	Cross‐sectional	PHC in Brazil	Data from 9273 phone teleconsultations during the period from September 2018 to July 2021 ordered by 3125 PHC nurses across all states of the country using TelessaúdeRS‐UFRGS	− There were 362 different reasons for ordering teleconsultations − The most frequent diagnostic codes were respiratory (25.9%), general and non‐specific (21.2%) and dermatological (21.2%) − 66.9% of the teleconsultations resulted in maintenance of the case in the PHC
3	Analysis of the Structure of Basic Health Units in Brazil to Conduct Telehealth Actions: A Comparison of Two Cross‐Sectional Studies [17] (2024)	To compare the ICT infrastructures in the basic health units in Brazil for telehealth actions along with the second and third cycles of the National Program for Access and Quality Improvement in Primary Care	Cross‐sectional	PHC in Brazil	Data from 22,021 basic health units assessed for availability of computers, cameras, sound equipment, microphones, printers, television and Internet	− There was an increase in the median number of ICT devices in the basic health units across all regions of Brazil − South, Southeast and Centre‐West regions had the highest median values in both cycles
4	User Experience Regarding Digital Primary Health Care in Santarem, Amazon: Evaluation of Patient Satisfaction and Doctor's Feedback [18] (2023)	To assess the feedback of patients and doctors on the use of telemedicine in outpatient care in Paisandú, a riverside district in Amazon	Cross‐sectional	PHC in a riverside district in Santarem, state of Pará	111 patients attending the basic health unit and feedback from a local doctor	− More than 95% of the patients reported being satisfied with the service; 87.4% had previous experience with videoconference, and 76.6% reported that their demand had been fully met − It was verified that 99.1% of the care services had some problem with audio or video
5	TeleOftalmo: Strategy to Expand the Offer of Ophthalmologic Telediagnostics for Primary Health Care in the Southern Brazil [19] (2022)	To describe 30,315 telediagnoses in ophthalmologic patients in the PHC and resolution of this service	Cross‐sectional	PHC in the state of RS	Data from 30,315 telediagnoses in ophthalmologic patients in the PHC who attended eight remote units of the TeleOftalmo Project between 2 January 2018 and 31 December 2020	− Refractive errors were the most prevalent diagnosis Presbyopia was the most frequent ocular disease in adults (65.4%) and elderly (64%), followed by cataract (41.3%) and suspected glaucoma (10.6%) in elderly − 70.5% of the patients had their ocular complaints fully resolved, with no need for referral to an ophthalmologist
6	Incorporation of Telehealth in Primary Health Care and Associated Factors in Brazil [20] (2022)	To analyse the use of the Brazilian Telehealth Network Program in the context of PHC in Brazil by identifying related factors	Cross‐sectional	PHC in Brazil	Data from 29,756 healthcare teams who participated in the second cycle of the National Program for Access and Quality Improvement in Primary Care	− Teleeducation for health professionals was the most frequently used modality (69.5%) − The highest rates of telehealth use were found in municipalities with up to 30,000 inhabitants in the South and Southeast regions − Institutional support had a positive impact, increasing the rate of telehealth use by 40%
7	Telehealth in Oral Medicine: Report of an Experience from Public Health Care in a Southern Brazilian State [21] (2022)	To analyse the asynchronous telediagnostic service for oral lesions by using the Brazilian Telehealth Network Program in the state of Paraná	Cross‐sectional	PHC in the state of Paraná	162 teleconsultations evaluated by 44 dentists between January 2015 and December 2019	− Information was enough for diagnostic reasoning in 77.16% of the orders − The teleconsultant verified that 48.1% of the cases could be resolved in PHC units − Dentists considered that teleconsultation fully met their needs in 42% of the cases
8	National Analysis of Dental Teleconsulting of the Brazilian Telehealth Program [22] (2022)	To describe regional differences in dental teleconsultation provided by the Brazilian Telehealth Network Program	Cross‐sectional	PHC in Brazil	Data from 2703 teleconsultations in 2019	− The Southeast region had the highest demand (49.1%) − 90.1% of the dentists reported to be satisfied − Referrals to other services were avoided in 66.8% of the cases
9	Quality of Telehealth Services in Primary Care in the State of MG [23] (2021)	To assess the quality of the telehealth services in PHC provided in the state of MG according to the ISO standard 13131	Cross‐sectional	PHC in the state of MG	Data from 385 municipalities of the state of MG obtained by stratified sample	− 64% of the municipalities had records of telehealth activities − 51% of the municipalities had high‐quality telehealth services − There was no association between quality of telehealth and quality of care; only the dimensions quality and risk management were shown to be associated with quality care
10	Telemedicine as Support for Primary Care Referrals to Neurologists: Decision‐Making between Different Specialists when Guiding the Case Over the Phone [24] (2021)	To describe the most frequent doubts reported by PHC doctors during teleconsultations, to compare decision‐making between neurologists or other specialists when guiding cases over the phone, and to assess how a teleconsultation service among doctors can reduce the number of referrals to other services	Cross‐sectional	PHC in Belo Horizonte, Porto Alegre and Distrito Federal	Data from 1687 teleconsultations performed from January 10 to 2 December 2019	− Epilepsy was the most frequent doubt among doctors − After discussion via telemedicine, 25% of the referrals were avoided − Decision‐making was not influenced by the speciality of the teleconsultant physician
11	Impact of Teleconsultations on the Conduct of Oral Health Teams in the Telehealth Brazil Networks Programme [25] (2020)	To assess the impact of teleconsultations on the clinical qualification of the oral health teams in PHC units of the state of Rio Grande do Sul in the Brazilian Telehealth Network Program	Cross‐sectional	PHC in the state of RS	285 dentists and 132 oral health assistants of family health teams	− Oral health teleconsultation reduced the number of referrals to other PHC services by more than 45% − Teleconsultation was shown to be a feasible model of professional qualification aimed at improving oral care in the PHC
12	The Use of Telemedicine to Support Brazilian Primary Care Physicians in Managing Eye Conditions: The TeleOftalmo Project [26] (2020)	To determine whether teleophthalmology can help doctors evaluate and treat ocular problems and verify which clinical conditions can be treated in PHC settings	Cross‐sectional	PHC in the state of RS	Data from 8142 patients with eye health record	− Teleconsultation enabled PHC doctors to resolve more than 1/3 of the ocular complaints − Refractive errors had high rates of resolution, with reduction in the number of referrals to specialized care
13	Factors Associated with the Non‐Use of Telehealth Consultancy by Physicians of the Family Health Strategy [27] (2019)	To assess rates of use and factors associated with non‐use of teleconsultation service by doctors in the Family Health Strategy program in the north of the state of MG	Cross‐sectional	PHC in the north of the state of MG	385 doctors of 73 municipalities in the north of the state of MG	− 55.8% of the participants did not use the teleconsultation service − Variables associated with non‐use: unavailability of computers with internet, lack of information on the service and the absence of training for use of teleconsultation
14	The Majority of Skin Lesions in Pediatric Primary Care Attention Could Be Managed by Teledermatology [28] (2019)	To assess the rate of lesions in paediatric patients who could be treated by using teledermatology in the PHC	Cross‐sectional	PHC in the city of São Paulo	Data from 6879 people and 10,126 skin lesions obtained in the period between July 2017 and July 2018	− 63% of the lesions were evaluated by using telescreening without needing on‐site consultation − Acne was the most significant problem among participants aged 13–19 years old, followed by atopic dermatitis, nevus and warts
15	Use of Maranhão Telehealth Program as a Tool to Support Continuing Health Education [29] (2018)	To assess the use of services provided by the Maranhão state telehealth programme as a tool to support continuing health education for PHC professionals	Descriptive	PCH in the state of Maranhão	In 2015, a total of 6075 teleconsultations were made by 261 basic health units and 313 health teams. In 2016, a total of 7901 teleconsultations were made by 270 basic health unit and 356 health teams. In total, 13,976 teleconsultations	− The average use of teleconsultations and monthly rate of use *per* municipality and basic health unit were higher than those found elsewhere − Nurses and community health agents were those who most used the services − Among the professionals who evaluated the service, more than 80% reported having their doubts solved
16	Telediagnosis of Oral Lesions in Primary Care: The EstomatoNet Program [30] (2018)	To summarize the experience with EstomatoNet programme, which is a telediagnostic tool for PHC dentists and physicians in the south of Brazil	Cross‐sectional	PHC in the state of RS	71 dentists and 18 physicians in the PHC	− Teleconsultants recommended referrals to specialists in 42.9% of the cases, total biopsy in 23.6% and follow‐up in 16.2% − After using this service, referrals to on‐site consultation decreased from 96.9% to 35.1%
17	Belo Horizonte Telehealth: Incorporation of Teleconsultations in a Health Primary Care System [31] (2018)	To analyse opinions on obstacles, difficulties and suggestions regarding the use of teleconsultations	Qualitative	PHC in Belo Horizonte, state of MG	30 health professionals and managers in the PHC	The main difficulties reported were related to the use of the system, inadequate response to teleconsultation demands, problems with infrastructure, lack of easy use of the system, difficulty in implementing the system within the organizational culture and lack of managerial support − Neurology, cardiology, endocrinology and angiology were the most required specialties
18	Analysis of the Asynchronous Dental Teleconsulting of Telehealth Brazil Networks in MG [32] (2018)	To assess the asynchronous dental teleconsultation of the Brazilian Telehealth Network Program in the state of MG	Cross‐sectional	PHC in the state of MG	Data from 3920 teleconsultations performed in the period from July 2015 to July 2017	− The most common teleconsultations involved specializations, such as pathology/stomatology (19.0%) − Most of this service was ordered by dentists (93.3%)
19	Providing Telehealth Services to a Public Primary Care Network: The Experience of RedeNUTES in Pernambuco, Brazil [33] (2016)	To report an experience with the RedeNUTES in the state of Pernambuco, Brazil	Descriptive	PHC in the state of Pernambuco	Data from 106 municipalities, totalizing 411 telehealth points and 862,500 people attending the basic health unit	− Teleeducation for health professionals and teleconsultation were the most used modalities − Nurses were the professionals who most used teleconsultation services (36%) − Telediagnosis for electrocardiogram has been increasingly used − The rate of satisfaction was 89%, and 68.5% of the professionals changed their planning to referrals to specialists
20	Telehealth Support for Primary Health Care in Remote Municipalities in the State of MG, Brazil [34] (2014)	To analyse the use, effectiveness and usefulness of teleconsultation services provided by the Telehealth Network of MG, in which teleconsultation and telediagnosis in cardiology are offered to 821 basic health unit	Descriptive	PHC in the state of MG	Data from 47,689 teleconsultations were performed between April 2007 and December 2012	Nurses (53.2%) and doctors (34.3%) were the professionals who most used teleconsultations − The main doubts were about pharmacological treatment, non‐pharmacological treatment and aetiology − Teleconsultations avoided potential referrals in 80% of the cases, and 94% of the health professionals reported satisfaction with the service

Abbreviations: ICT, information and communication technology; MG, Minas Gerais.

*Source:* Elaborated by the authors.

It was found that there was a lack of studies addressing experiences with remote consultation for follow‐up and education of patients with non‐communicable chronic conditions, as well as teleservice provided by multi‐professional teams. It was observed that the studies addressed the role of core teams of PHC professionals involving physicians, nurses and dentists. No study on teleconsulting for mental health was found either, a frequent demand in the PHC.

## 4 Discussion

The studies identified experiences with telehealth in the Brazilian PHC. Remote contact between health professionals for diagnosis, improvement of clinical practices and resolution of doubts was frequently reported. Nevertheless, there is a lack of investigations on the direct interaction between patients and health professionals by means of teleconsultation. In addition, no study was found that addressed the interface of telehealth with attributes derived from PHC, such as cultural competence as well as family and community orientation.

These attributes were interpreted on the basis of the classical framework proposed by Barbara Starfield [33], which defines PHC through essential attributes such as access, longitudinality, comprehensiveness and coordination, as well as derived attributes, including family and community orientation and cultural competence, widely adopted in international literature and aligned with the conceptual organization of PHC in the SUS. This gap highlights the need for future studies to explore telehealth not only as a technological tool but as a strategy capable of strengthening core PHC attributes within the SUS. This distinction highlights that telehealth in Brazilian PHC has been predominantly used as a support tool among professionals rather than as a direct care modality for patients.

PHC is internationally acknowledged for carrying out health actions, both individually and collectively, capable of meeting several needs. It was found that to provide full healthcare, these studies reported results of telehealth services for consultation between health professionals to promote clinical and care management. In fact, complex cases in the PHC have been solved by using this modality of telehealth, with reduction of unnecessary referrals to specialized care [34].

Although these articles have shown a scenario of telehealth in different regions of the country, there were differences between the Southeast and South regions regarding its use. It is important to highlight that the evidence identified in this review is predominantly concentrated in the Southeast and South regions, especially in the states of MG and RS, which limits the generalizability of findings to the entire Brazilian territory. This was also observed in other states of the country. The external assessment results of the National Program for Access and Quality Improvement in Primary Care (PMAQ‐AB), obtained from Modules I and II of the first (2012), second (2014) and third (2018) cycles, showed that there was an increase in the use of telehealth by PHC teams between 2012 (12.7%), 2014 (27.7%) and 2018 (54.6%). However, regional discrepancies remain, as the lowest rates of telehealth use were observed in the North and Northwest regions [11].

Telehealth has been shown to be feasible for managing non‐communicable chronic conditions in the PHC. By means of several functions, this model of care not only alters the way how telehealth services are provided but also can positively impact the person‐centred care and clinical outcomes. In the present review, no impact study was found. However, a systematic review with meta‐analysis assessed the use of telehealth in a PHC for management of cardiovascular risk factors among people with systemic arterial hypertension and Type 2 diabetes. It was verified that there were short‐term benefits, which suggests a combination of teleconsultation with educational actions [35].

Health education is one of the strategies for PHC to ensure continuity of care. Nevertheless, there is a shortage of studies addressing telehealth in the context of education for population. A scope review on the use of telehealth during the COVID‐19 pandemic reported such experiences in different countries, but only a few studies cited the issue of education. In South Africa, it was reported that education on health is limited to the academic context. In Canada, this theme emerges with the objective to implement training on technologies to improve the provision of healthcare services. In the United States, education through telehealth appears as a facilitator of implementation. In Germany, the theme came up when education of health professionals on the use of ICTs was addressed. In Hungary, telehealth was shown to facilitate the prescription of medicines [36].

In the present review, it was detected that telehealth involved core teams of FHS professionals, such as physicians, nurses and dentists. Nevertheless, one can highlight the importance of engagement and potentialities of a multi‐professional team in the provision of services through ICTs [37]. A Brazilian web survey conducted with diabetic patients in 2020 showed that about 40% of the participants used teleconsultation with physicians, nutritionists, pharmacists, nurses, psychologists and other health professionals during the pandemic period [38]. Therefore, there is a need for studies demonstrating multi‐professional actions in the PHC, considering complexity of the cases and singularity of the individuals.

Telehealth has consolidated itself as an essential tool for PHC in Brazil, thus increasing access to healthcare services and favouring integration of care, especially in remote and vulnerable areas. However, its expansion also raises challenges related to digital governance, data reliability and the management of health misinformation, which have been increasingly discussed in the context of digital public health [39]. Recent studies pointed out that telehealth provides real‐time clinical support and enables collaboration between health professionals, which helps overcome geographical barriers and enables quality care to isolated populations, such as riverside communities and indigenous people [40]. The Brazilian experience in the PHC showed that telehealth not only improved access to care but also strengthened the health system's capacity for response in difficult‐to‐reach areas, such as health services provided in the Amazon region [41].

In situations of social and geographical vulnerability, telehealth can contribute to the continuity of care and promote a more equal care, including follow‐up of chronic diseases. By using mobile devices and remote monitoring technologies, health professionals can quickly adjust treatments, thus contributing to the prevention of severe complications and avoiding unnecessary hospitalizations [42, 43].

Among the limitations of the present review, one can cite the absence of intervention studies, which would allow a broader discussion about telehealth in the PHC. Despite this, one believes that these observational studies allow us to outline a profile of ICT‐mediated actions in the country and discuss the need to strengthen the digital inclusion within the PHC policy without affecting the characteristics of services accounting for the coordination of healthcare networks.

## 5 Conclusion

Although telehealth initiatives have been reported in different regions of the country, the available evidence is concentrated in specific states, particularly in the Southeast and South regions; there are distinctions, especially regarding infrastructure. Teleconsulting and telediagnosis were found to be the most used modalities, but there is a lack of studies on multi‐professional teams. These strategic proposals involve a telehealth agenda for health professionals, as well as a stimulus for person‐centred clinical reasoning and a critical thinking which considers the attributes proposed for PHC.

## Author Contributions

**Márcio Cristiano de Melo**: conceptualization, investigation, funding acquisition, writing – original draft, writing – review and editing, validation, methodology, visualization, software, formal analysis, project administration, resources, supervision, data curation. **Elizabeth Regina de Melo Cabral**: conceptualization, investigation, funding acquisition, writing – original draft, writing – review and editing, visualization, validation, methodology, formal analysis, data curation. **Rinaldo Eduardo Machado de Oliveira**: conceptualization, investigation, funding acquisition, writing – original draft, writing – review and editing, visualization, validation, methodology, project administration, formal analysis, software, data curation, supervision, resources. **Ivana Daniela César**: conceptualization, investigation, writing – original draft, writing – review and editing, visualization, validation, methodology, formal analysis, data curation. **Brenda Leandro dos Santos**: conceptualization, investigation, funding acquisition, writing – original draft, writing – review and editing, visualization, validation, methodology, formal analysis, data curation. **Lívia Maria Ferrante Vizzotto Consoli**: conceptualization, investigation, funding acquisition, writing – original draft, writing – review and editing, visualization, validation, methodology, formal analysis, data curation.

## Funding

This study was financed in part by the Coordenação de Aperfeiçoamento de Pessoal de Nível Superior—Brasil (CAPES)—Finance Code 001.

## Conflicts of Interest

The authors declare no conflicts of interest.

## Data Availability

Research data can be requested from the corresponding author.
